# To Avoid and to Prevent Consumption

**Published:** 1895-05

**Authors:** 


### TO AVOID AND TO PREVENT CONSUMPTION.

THE Ohio State Board of Health has promulgated (*Columbus Medical Journal*, July 24th,) the following rules on the subject of tuberculosis. We print them in full, as they are worthy of careful study.

How to prevent consumption: 1. In the house the expectorations of a consumptive patient should be received on bits of old cloth or Japanese paper and be burned at once or received in cuspidors or spit cups containing a solution of:

| | | |
|---|---|---|
| ℞ | Corrosive sublimate | ʒ j. |
| | Hydrochloric acid | ℥ ij. |
| | Water | 1 gallon. |

Label, "Poison."

2. The clothing and bedding of the patient should be laundried separately and thoroughly boiled.

3. Sweeping should be done with a dampened broom or with wet tea leaves or sawdust on the floor, and the dust removed from the furniture, etc., with a cloth wet with the disinfectant solution.

4. Dishes, glasses, cutlery, etc., used by the patient should be scalded before using again.

5. It is better for the patient and safer for others that he sleep in a room alone and especially in a bed to himself.

6. The disease may be transmitted by kissing, especially kissing upon the mouth.

7. Admit an abundance of pure air and sunlight to the patient's room.

8. If the house is damp use proper means to secure dry foundation walls and basement.

9. On the street the patient should either use an expectoration flask (many such are made) or cloths or paper, to be burned as soon as possible. If a handkerchief must be used, place it in boiling water or in a disinfectant solution before the expectoration can dry.

To avoid consumption: 1. Eat meat cooked well done, as this will destroy the germ. Boiling will destroy the germs in milk, and young children, who are especially prone to tuberculosis of the bowels, should be given only boiled milk.

2. A mother with consumption should not suckle her child, as she may infect it through her milk.

3. Do not move into a house or sleep in a room in which a person has died of or been sick with consumption until it has been properly disinfected.

4. Avoid as far as possible occupying any length of time with a consumptive person a badly ventilated room, car or vessel.

5. If a tendency to the disease has been inherited, be specially guarded against all sources of infection. In addition, select an out-door occupation as free as possible from dust; use every means to secure a good physical development, particularly of the chest and lungs; select a dry soil for habitation and have living rooms freely ventilated and well exposed to direct sunlight.

6. In selecting a mate in marriage choose one free from any inherited scrofulous or tubercular taint.

We trust all persons reading this circular will aid in disseminating the information it contains. It is only by rousing the public to a realizing sense of the fact that consumption is communicable and preventable that we may hope to stay the ravages of this disease, which alone slays more than all the other contagious diseases combined.

